# A New Environmental Dependency Syndrome Occurring With Frontotemporal Lobe Degeneration: Hypervisual Illusory Spread Syndrome

**DOI:** 10.7759/cureus.18119

**Published:** 2021-09-20

**Authors:** Michael Hoffmann

**Affiliations:** 1 Internal Medicine, University of Central Florida College of Medicine, Orlando, USA; 2 Neurology, University of Central Florida College of Medicine, Orlando, USA

**Keywords:** emotional dysregulation, progressive aphasia, visual illusions, visual symptoms, frontotemporal syndrome

## Abstract

A 59-year-old man presented with non-fluent aphasia and behavioral variant frontotemporal lobe degeneration (FTD), volunteered unusual visual symptoms that were best described as illusory visual spread (the image spreading over a larger area). This type of visual hyperfunction, related to the palinopsia syndromes, has not been reported in association with FTD. The syndrome may be best understood in terms of a visual variant of the environmental dependency syndrome, akin to the verbal variant of forced hyperphasia syndrome.

## Introduction

Frontotemporal disorders present with an extensive array of syndromes, including many frontal lobe presentations as well as motor, language and memory disorders. Visual disorders have rarely been documented and to date limited to oculomotor disorders and visuospatial memory recall. As the occipito-frontal fasciculus is the longest and also one of the largest fiber tracts in the human brain, visual disorders with frontal pathologies may be important in documenting the range of symptoms, be important in monitoring progression or deterioration and in the understanding the etiopathogenesis [1].

## Case presentation

**Figure 1:**
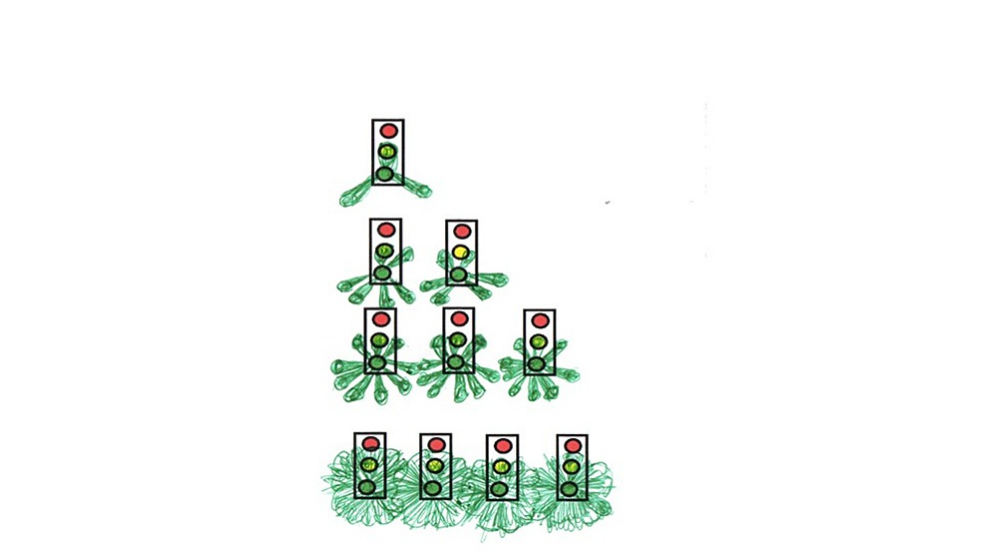
Looking at traffic lights particularly at night, one, two, three and four lights and the cone with circle at end dispersion effects.

**Figure 2:**
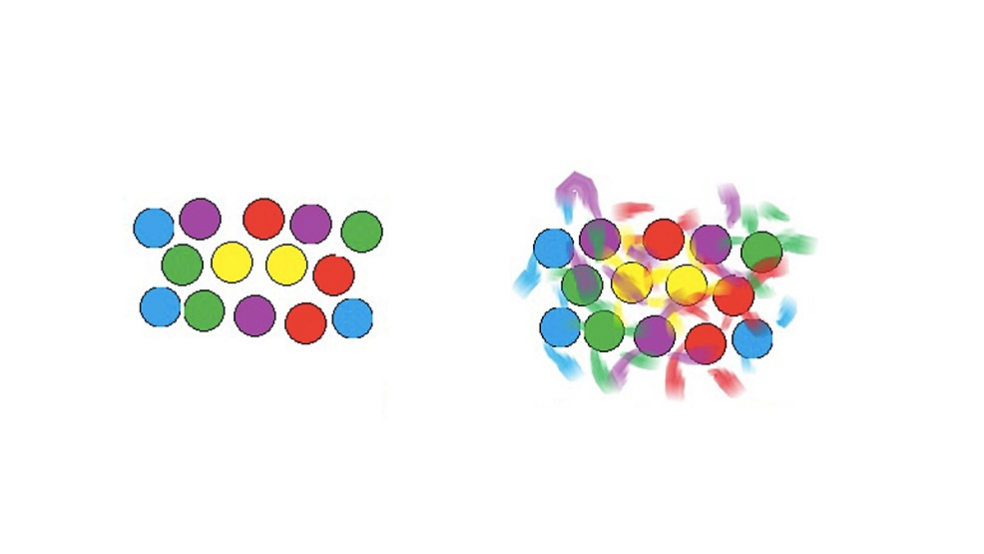
Cognitive game with different colored bubbles. Similar effect to traffic light but less organized dispersion and loss of shape of the lobes. After looking at the bubbles for several seconds, this illusion takes effect.

**Figure 3:**
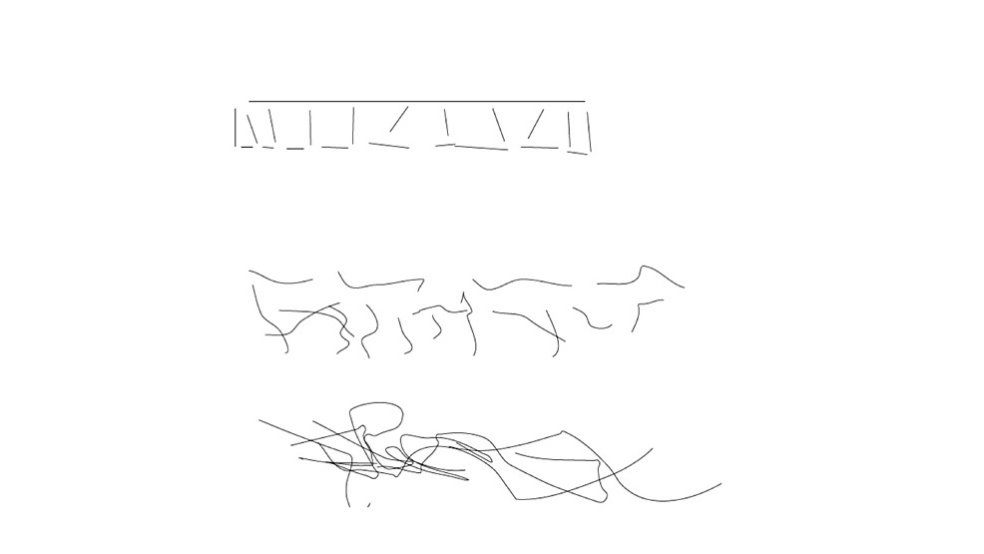
When looking at two parallel horizontal lines the following happens: The lines start wandering and curving after 2-3 seconds (top image) Last, they become a hopeless mess (middle image). Looking at it for more than 6-7 seconds, a headache emerges (lower image).

We report the case of a 59-year old, right-handed man with a three-year history of speech impairment and emotional lability. His educational level was notable for a six-year college education culminating in an engineering degree. His spouse had noted hesitancy in speech, difficulty with formulating words but with a marked fluctuation. Among his symptoms volunteered by himself and corroborated by his wife, he described several different unusual visual experiences. Because of these unusual features, he was asked to draw and describe in his own words what he perceived (Figures 1, 2, 3), “draw exactly what you see”. Of note is that he appeared to be a good artist. The comments are his direct quotes at time of constructing the images. For example, while driving at night in particular, he had noted “an extension of the traffic light color in the form of triangular cones of the green light, always evenly spaced at specific positions, that he depicted at 12, 4:30 and 7:30 o’clock. This would be amplified by the number of lights” that were strung across the intersection, depending on the size of the roads. With four or more lights, there was extensive overlap but they still appeared as cones but very much blurred together (Figure 1). Other illusions occurred with a game he played comprising of different colored circles that would also take on a type of illusory spread of the colors emanating off the discs (Figure 2). In yet another example he reported when gazing at parallel lines, after a minute or so appear progressively more convoluted until they had the appearance of “spaghetti” (Figure 3). Additional symptoms included hearing music and voices three to four days per week lasting several minutes at a time. Sometimes it occurs many times during the day and night and is more pronounced when tired. He is no longer able to rotate objects in three-dimensional space. Other symptoms volunteered by his wife included irritability, rarely anger attacks and proneness to crying and much less often inappropriate laughing. The overall format of the interview proceeded with open-ended questioning, he was encouraged to both explain and draw the images. Interviews were both individual and together over a period of approximately 1.5 hours and recorded in the standard clinic electronic record. Specifically, he was not abusing substances, a non-smoker and not prone to alcohol abuse. He was not on neuropsychiatric, sedative or hypnotic medications. 

Significant past medical history was notable for a boxing career of eight years, during which he incurred six “knock-outs” as well as two blast traumatic brain injuries. He did not suffer from migraines or other headache syndromes and no other notable past medical or surgical history was evident. Specifically, he was not abusing substances, was a non-smoker, not prone to alcohol abuse and was not using psychiatric, sedative or hypnotic medications. 

Examination

General examination was unremarkable, with a normal body mass index and neurological examination notable only for hyposmia and mild peripheral neuropathy evidenced by absent ankle reflexes. Specifically, visual acuity was recorded at 20/25 for each eye, external ocular movements full without nystagmus and otherwise devoid of any ophthalmological testing abnormality. Cognitive screening evaluation with a Montreal Cognitive Assessment (MOCA) (22/30) examination was notable for degree of dyscalculia with for serial 7’s 0/5, but when aided by writing he correctly obtained 5/5, orientation was 5/5 and word list generation with “F” 12 words in one minute. Memory testing with 5 words at 5 minutes, he recalled 3/5 correctly. There was significant dysfluency, that fluctuated with tiredness associated with mild dysarthria. Additional bedside testing included an abbreviated Pyramid and Palm Test with 6/10 items correctly associated. Visuospatial function testing for reproducing two-dimensional figures was intact (drawing a daisy flower) and impaired for three-dimensional, reproducing a Necker cube. Subtests of the Visual Object and Space Perception Battery Silhouette subtest was impaired with 0/3 figures correctly interpreted. Screening testing for achromatopsia with the Western Aphasia Battery (WAB) color test he named 6/6 correctly, the WAB picture test 3/7 figures identified and an abbreviated Poppelreuter figure test item, 0/6 were identified.

Neuropsychological

Neuropsychological testing was performed on two separate occasions over a three-year period with similar findings at both visits for the domains of memory (visual, verbal), attention, executive function and speed of information processing. A screening Mini-Mental State Examination (MMSE) at the initial visit was 25/30. In summary, testing revealed relatively mild impairments in terms of borderline executive dysfunction, mild speed of information processing deficit, memory encoding and retrieval impairment, depression (BDI 13/63) and digit reversal impairment. The neuropsychological findings were interpreted as supporting a somatoform disorder. Tests and subtests included the Boston Naming Test, WRAT 4, WAIS IV Digit Span, Serial Subtraction, Trail Making Test, WMS-III Logical Memory Test, Hopkins Verbal Learning Test Revised, Brief Visuospatial Memory Test Revised, Verbal Fluency Tests, DKEFS Color Word Tests, Test of Memory Malingering (TOMM), Beck Depression Inventory-II, Beck Anxiety Index and PTSD check list. 

Behavioral testing

Frontal Behavioral Inventory (FBI) Test 

He scored highest (3) in the domains of apathy, emotional flatness (indifference), inflexibility, disorganization, loss of insight, inappropriateness, poor judgment and impulsivity and irritability. A score of 2 was given for inattention, logopenia, hyperorality and incontinence. A score of 1 was given for aspontaneity, aphasia and verbal apraxia and personal neglect. The total FBI score 36 where > 27 is supportive of a frontotemporal syndrome. 

Frontal Systems Behavior Examination (FRSBE) [2]

The T score for abulia was 97, disinhibition 74, executive dysfunction 93 with a total score 97. Age, gender and education normed score evaluated by family or self and scored in T scores where > 60 is abnormal.

Involuntary Emotional Expression Disorder (IEED) Assessment

The Center for Neurologic Study-Lability Scale (CNS-LS) for pseudobulbar affect [3].

A patient scored quantitative measure with a score of > 13 is supportive of IEED. Our patients score was 15.

Neuroimaging

**Figure 4:**
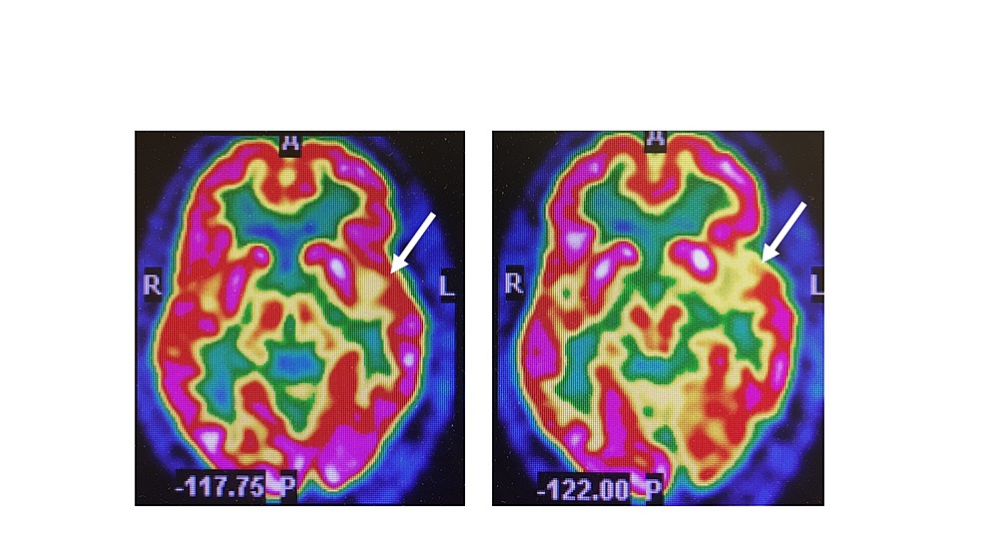
PET brain scan revealed posterior left inferior frontal lobe and anterior insula hypometabolism (white arrows).

A 3 Tesla MRI General Electric scan with imaging sequences including T1, T2, FLAIR, GRES with 8cc Gadavist IV contrast administered. The diffusion tensor image (DTI) interpretation included color-coded fractional anisotropy DTI sequences and examined visually for asymmetry and interruption of the white matter myelinated fiber tracts. Absolute selected FA values were recorded in five major tracts in each hemisphere, with a lower limit of normal set at 0.40. The structural MRI brain revealed no atrophy but minimal leukoaraiosis. Diffusion tensor imaging was reported normal. Functional brain imaging with positron emission tomography (PET) brain scanning was subsequently performed. With the patient rested in a quiet low-lit room after 17.85 mCi of F-18 FDG was injected intravenously. The pre-injection glucose was 85 mg/dl. PET and non-contrast computerized tomography (for attenuation correction and anatomic localization) were acquired sequentially of the head and fusion and MIP images were constructed. The PET brain scan was abnormal and depicted in Figure 4.

Electrophysiological Testing

A standard EEG and visual evoked responses were within normal limits.

## Discussion

Our patient presented primarily with a non-fluent aphasia variant and behavioral variant frontotemporal syndrome (bv-FTD) according to the Rascovsky criteria [4]. The bv-FTD incorporated both abulic and frontal disinhibition syndromes reflecting involvement of the mesial anterior frontal and medial orbitofrontal circuitry. The visual phenomena reported by the patient are remarkable in that they took the form of a type of illusory visual spread of certain objects that he encountered and depicted graphically himself, with remarkable skill. Similar phenomena have been described previously by Critchley but only in the context of posterior lesions, termed illusory visual spread, that were attributed to be part of the spectrum of palinopsias [5]. In brief, palinopsias refer to the illusion of the persistence of an image in the environment after egress of the original object. A similar syndrome has been described in association with migraine attacks, albeit with some differences [6]. Visual perseverations may be regarded as a generic term for a number of different kinds of visual illusions. They may take the form of visual illusions with persistence both in time and the abnormal spatial spread of a visual percept. Based on the time interval, these may be classified as immediate palinopsias, true palinopsias (lasting minutes to hour) and hallucinatory palinopsias (maybe days to weeks between object viewing and illusion) by Kölmel [7]. The illusion of visual spread may be a type of visual perseveration in space. Such visual illusions need to be interpreted in the context of other syndromes seen in association with the FTD conditions such as novel artistic expertise. Right hemisphere-related exceptional art and music compositions after left anterior dysfunction have also been described by Miller [8] and Schott [9]. Also relevant is the unique visuospatial capabilities related jig-saw puzzle expertise with FTD semantic variant, reported by Green [10]. Other recently reported, right hemisphere predominant, visuospatial creativity syndromes include architectural brilliance that has also been described in association with FTD. The example of architectural aptitudes of King Ludwig II was reported by Förstl et al, who designed three castles, the most notable being Neuschwanstein also known as the “Disney” castle [11].

New found artistic ability and creativity have been attributed to ‘release mechanisms’ ascribed to the consequences of diaschisis, which follow left hemisphere lesions, rendering an upsurge of function within the right hemisphere. Both intra-hemispheric and inter-hemispheric hodologic effects have long been reported, substantiated by PET scanning [12]. The hodotopic approach to understanding brain lesion effects refers to the lesion itself inducing either hypofunction or hyperfunction or hodological effects may take the form of disconnection either a hypoconnection or hyperconnection [13]. More recently network analyses in relation to right hemisphere neglect patients after stroke have supported more widespread cortical and subcortical alterations [14]. In Alzheimer’s disease network alterations may predate clinical symptoms by decades [15]. Presumably such mechanisms also occur in FTD syndromes and may provide a pathophysiological explanation for the syndrome reported in our patient. Right hemisphere hyperfunction may also be seen in Savant syndromes with the purported mechanism thought to be the mechanism of paradoxical functional facilitation [16]. Usually 5 prodigious abilities are noted including music, art, visuospatial skills, mathematics and calendar counting may be appreciated. However, there are many other less common abilities such as hyperlexia, language (polyglots), vision, synesthesia, smell, time appreciation, navigation and statistical skills that may also emanate after left hemisphere hypofunction due to whatever reason [17]. More recently a pathophysiological explanation may be found in network analysis literature. Neuronal network activities supporting cognition are altered decades before clinical disease becomes apparent. Such synchrony deficits that include hypersynchrony such as gamma oscillation (30-150 Hz) and interneurons are thought to be responsible for various cognitive syndromes and dysfunctions.

It is intriguing that in addition to the “visual hyperfunction” reported by our patient he also experienced frequent musical hallucinations, underscoring the right hemisphere musical capabilities and propensities. The forced hyperphasia syndrome is regarded as a verbal variant of environmental dependency syndrome that has been described with a left hemisphere lesion [18]. Our patient’s syndrome may be most appropriately understood in terms of a visual variant of the environmental dependency syndrome (EDS), akin to the forced hyperphasia syndrome verbal variant. This may be viewed as new visual variant subtype of environmental dependency syndrome, presenting with the illusory visual spread. As regards the etiopathogenesis, he presented with predominant non-fluent aphasia variant of frontotemporal lobe degeneration with superadded behavioral variant of frontotemporal lobe syndrome attributed to recurrent trauma with a possible CTE type of pathology. This was supported by the PET brain scan that revealed left inferior frontal gyrus, minimal left basal ganglia and insula hypometabolism as reported by Gorno-Tempini et al [19]. The syndrome presented, is regarded as a newly appreciated field-dependent behavior syndromes first described by Lhermitte [20]. A proposed classification by the author includes elementary forms such as imitation behavior, utilization behavior and environmental dependency syndromes. More complex forms include verbal subtypes (echoing approval, forced hyperphasia), visual subtype (hypervisual illusory visual dispersion), behavioral subtypes (Zelig syndrome, echopraxia to television) and motor subtypes (Tourette’s syndrome, obsessive-compulsive syndrome, exaggerated startle response). 

## Conclusions

In conclusion, a patient with non-fluent aphasia and behavioral variant frontotemporal lobe degeneration (FTD), volunteered unusual visual symptoms that were best described as illusory visual spread (the image spreading over a larger area). This type of visual hyperfunction, related to the palinopsia syndromes, has not been reported previously in association with FTD. The syndrome may be best understood in terms of a visual variant of the environmental dependency syndrome, akin to the verbal variant of forced hyperphasia syndrome. From a neuro-archeological point of view, the most expansive cerebral networks, the frontoparietal systems, include both the all-important working memory (WM) circuitry and mirror neuron (MN) circuitry. The MN circuit and a third major network, the frontal inhibitory circuit is pertinent in the pathophysiology of environmental dependency syndromes. The syndrome presented, may be interpreted in terms of the wider field-dependent behavior syndromes.
